# Molecular Autopsy in Asphyxia Deaths: Diagnostic Perspectives of miRNAs in the Evaluation of Hypoxia Response

**DOI:** 10.7150/ijms.79539

**Published:** 2023-04-24

**Authors:** Matteo Scopetti, Martina Padovano, Federico Manetti, Nicola Di Fazio, Davide Radaelli, Stefano D'Errico, Paola Frati, Vittorio Fineschi

**Affiliations:** 1 Department of Medical Surgical Sciences and Translational Medicine, Sapienza University of Rome, Rome, Italy.; 2 Department of Anatomical, Histological, Forensic and Orthopaedic Sciences, Sapienza University of Rome, Rome, Italy.; 3 Department of Medicine, Surgery and Health, University of Trieste, Trieste, Italy.

**Keywords:** Asphyxia deaths, Hypoxia response, Forensic pathology, Molecular autopsy, HypoxamiRs

## Abstract

The forensic investigation of asphyxia deaths still poses a challenge due to the need to demonstrate vital exposure to hypoxic insult according to high levels of evidence. The pulmonary effects of hypoxia are complex and the understanding of the mechanisms underlying the acute pneumotoxicity induced by hypoxia is still incomplete. Redox imbalance has been suggested as the protagonist of the main acute changes in pulmonary function in the hypoxic context. The development of knowledge in biochemistry and molecular biology has allowed research in forensic pathology to identify some markers useful in immunohistochemical diagnostics of asphyxia deaths. Several studies have highlighted the diagnostic potential of markers belonging to the HIF-1α and NF-kB pathways. The central role of some highly specific microRNAs has recently been recognized in the complex molecular mechanisms involved in the hypoxia response; thus, several research activities are currently aimed at identifying miRNAs involved in the regulation of oxygen homeostasis (hypoxamiR). The aim of the manuscript is to identify, the miRNAs involved in the early stages of the cellular response to hypoxia, in order to characterize the possible implications in the forensic field of the determination of expression profiles. At present, more than 60 miRNAs involved in the hypoxia response with different expression profiles (upregulation and downregulation) have been identified. Despite the multiple and different effects on reprogramming following the hypoxic insult, the evaluation of the diagnostic implications of hypoxamiRs in the forensic field presupposes a specific treatment of the influences on HIF-1α regulation, cell cycle progression, DNA repair, and apoptosis.

## Introduction

The forensic investigation of asphyxia deaths still poses a challenge due to the need to prove vital exposure to hypoxic insult according to high levels of evidence. Historically, the study of pulmonary histomorphological changes resulting from asphyxia has been considered of great importance for diagnostic purposes. However, the correlation of histopathological findings with the diagnosis of asphyxia death requires an accurate assessment of any concomitant conditions such as alterations in the relationship between ventilation and perfusion attributable to age, comorbidities, and exposure to smoke. Therefore, over the years, constant research has been aimed at studying the molecular response to the decrease in oxygen tension.

Hypoxia represents a fundamental physiological stimulus capable of triggering adaptive and maladaptive responses in a wide variety of pathophysiologically relevant situations. The effects of hypoxia at the pulmonary level are complex and the understanding of the mechanisms underlying the acute pneumotoxicity induced by the hypoxic insult is still incomplete. Redox imbalance has been suggested as the protagonist of the main acute changes in lung function in the hypoxic context, in particular as regards hypoxic vasoconstriction, endothelial dysfunction, edema, and lung inflammation. Growing evidence suggests that hypoxia is capable of causing severe mitochondrial oxidative stress in the lungs by increasing the production of ROS in complexes I and III of the electron transport chain 1. Pulmonary exposure to low oxygen tensions also favors the increase in the production of reactive oxygen species (ROS) by the enzymes NADPH oxidase, xanthine oxidoreductase and nitric oxide synthase (NOS) 2. In particular, the enzyme xanthine oxidase (XOS) has a proven role as a ROS generator in response to low oxygen concentrations, probably representing one of the most important sources of reactive species. A further source of ROS is constituted by NADPH oxidase (NOX), and in particular by the NOX-4 isoform, which is abundantly represented in the lungs. Finally, it was observed that NO synthase (NOS) is involved - through the production of nitric oxide - in several physiological (vasodilation and bronchodilation) and pathological (inflammation and oxidative damage) processes; specifically, the inducible isoform of the enzyme (iNOS) - activated in a hypoxic context by NF-κB, HIF-1α, and ROS - determines the production of reactive species responsible for the depletion of antioxidants.

The development of knowledge in the field of biochemistry and molecular biology has enabled research in forensic pathology to identify some molecular markers of response to hypoxic insult useful in immunohistochemical diagnostics of asphyxia deaths. Precisely, several scientific contributions have highlighted the diagnostic potential of markers belonging to the molecular pathways of HIF-1α and NF-κB.

The central role of some highly specific microRNAs (miRNAs) in the context of the complex molecular mechanisms involved in the response to hypoxia has recently been recognized. In parallel, a role of miRNAs as emerging diagnostic biomarkers of the cellular response to low oxygen tensions has been established, so much so that several research activities are currently aimed at identifying the miRNAs involved in the regulation of oxygen homeostasis (hypoxamiRs) 3.

Despite the evidence generated, currently, the molecular biology of the pulmonary response to hypoxic insult requires further investigation aimed at clarifying - among other things - the actual diagnostic usefulness in the forensic field. The majority of the studies carried out so far have been conducted on animal models and have been specifically directed to the evaluation of the expression profiles of hypoxamiRs in different pathological conditions such as pulmonary hypertension, myocardial infarction, hypoxic-ischemic encephalopathy, skin ischemic lesions, and neoplastic progression.

The main objective of the present study is the identification of a miRNA panel, whose expression in the lungs is induced by an acute hypoxic insult, to evaluate the actual possibility of using hypoxamiRs in the diagnosis of asphyxia deaths.

To this purpose, a literature search was carried out aimed at identifying the markers currently supported by scientific evidence. In the selection process, the molecular pathways currently studied in post-mortem diagnostics were also considered with the aim of conferring objectivity and facilitating efficacy evaluations.

## Forensic investigation of asphyxia deaths

The term "asphyxia" in its etymological meaning indicates the absence of the pulse, the cessation of the heartbeat, and therefore, by extension, the absence of respiratory acts or peripheral pulses with consequent respiratory block 4. Violent mechanical asphyxia includes the injurious forms characterized by a mechanical impediment to the airflow deriving from an external cause, of a mechanical nature, which is expressed with consistent energy, as well as in a sudden and rapid manner.

The main issues of interest in the diagnosis of asphyxia deaths concern the diagnosis of death, the comparative study of cadaveric phenomena for the estimation of the post-mortem interval (PMI), and the chronological diagnosis of the lesions as well as the differential diagnosis between vital phenomena and post-mortem. The medico-legal investigation of the chronology and vitality of the lesions requires the integration of different techniques and produces results that can be difficult to interpret, especially in cases of advanced decomposition and poor representation of the signs of the vital reaction.

At present, the integration of macroscopic evidence and microscopic data obtained from histological and immunohistochemical investigations allows in most cases the chronological and vitality diagnosis of the lesions.

The macroscopic diagnosis of mechanical asphyxia is based on the generic signs of asphyxia death and the presence of traumatic injury related to the application of injurious means 5-7. The findings relating to asphyxia death are devoid of exclusive pathognomonic significance as they can be found in the majority of sudden deaths regardless of etiology. Only the set of pathological findings, in view of the usual prudence in interpreting the results, can allow a diagnosis of asphyxia death, which must be definitively ascertained by objectifying the viability of the hypoxic insult and the lesions. As regards the signs of asphyxia death, the autopsy should be oriented to the search for external signs (cyanosis of the face, foamy material at the level of the respiratory orifices, submucosal hemorrhagic petechiae, protrusion of the eyeballs and tongue, traces of sperm at the level of the urethral orifice) and internal signs (emphysema and acute pulmonary edema, subserosal petechiae, diffuse visceral blood stasis, dilation of the right heart chambers, blood fluidity). Concerning traumatic injury, the different pictures of mechanical asphyxia are the expression of a peculiar typology and modality of action of energy which, in addition to generating the pathological picture described above, produces a characterizing lesion of primary diagnostic importance. The objectification of the pathognomonic signs of the various injurious actions on the corpse allows for differentiation of the various asphyxiogenic modalities.

The histopathological study of asphyxia deaths, as well as the autopsy one, is aimed at evaluating signs of asphyxia (cerebral edema, blood stasis, acute pulmonary edema, and emphysema), as well as traumatic lesions (traumatic alterations of the skin and soft tissues). For a long time, immunohistochemical techniques have been of great importance in the study of the vitality of lesions, since death is very rapid, affecting the tissue with alterations included in the early stages of the inflammatory response 8-10. Precisely, the immunohistochemical techniques have allowed to document the increased expression of molecules such as P-selectin, IL-15, CD15, and tryptase. On the contrary, the pro-inflammatory cytokines IL-1β and TNF-α are detectable by immunohistochemistry in lesions for which survival of more than 15 minutes has occurred; similarly, IL-6 significantly increases its concentration after 20 minutes from the action of the damaging agent; therefore, considering the narrow time interval between the traumatic insult and the asphyxia death, studies on animal and human models have limited their potential diagnostic applications. The scarcity of effectively usable immunohistochemical markers is justified by the rapidity of death resulting from the application of traumatic energy. In other words, given the precocity of the inflammatory response, only the markers involved in the initial reaction to the traumatic insult can be reliably used.

At present, the diagnosis of mechanical asphyxia still presents difficulties due to the poor specificity of the macroscopic signs and the limited expression of vitality related to the rapidity of death. Such a condition requires a reflection on the need to identify additional diagnostic means to be used in a complementary or, in some cases, substitute way. For this purpose, a joint evaluation of the inflammatory response, especially through the immunohistochemical markers just proposed, and of the hypoxic insult response through the study of the molecular pathway of HIF-1α could be useful. Undoubtedly, in addition to the methods outlined, a fundamental contribution to post-mortem investigations can be made by molecular biology through the study of miRNA expression profiles.

## Current evidence on hypoxamiRs

The synthesis of mature miRNAs represents a complex multiphasic process capable of undergoing the influence of low oxygen concentrations at practically all levels. While causing a reduction of about 50% in the levels of target proteins 11,12, miRNAs constitute an overall relevant component of the cellular response to hypoxia due to the coordinated and combined regulation of the targets.

HypoxamiRs are involved in the stereotyped rapid adaptive response to oxygen deprivation, even transient; precisely, miRNAs act rapidly at the post-transcriptional level to organize the response to environmental stress. Furthermore, such molecules coordinate a dynamic biological response based on gene regulation and - in particular - on transcription. Such a complex regulatory system is supported by significant molecular crosstalk aimed at effective communication between hypoxic signaling mechanisms and miRNAs (biogenesis and function).

Despite currently acquired knowledge, the precise mechanisms by which miRNAs carry out their multiple regulatory effects remain largely undefined. Further insights will derive from future research aimed at defining the complexity of hypoxic adaptive processes. In particular, the study activity will have to continue in the current direction toward the better characterization of the role of HIF and redox reactions in the regulation of miRNA biogenesis.

At present, more than 60 miRNAs involved in the hypoxia response with different expression profiles (upregulation and downregulation) have been identified. Despite the multiple and different effects on reprogramming following the hypoxic insult, the evaluation of the diagnostic implications of hypoxamiRs in the forensic field presupposes a specific treatment of the influences on HIF-1α regulation, cell cycle progression, DNA repair, and apoptosis (Table 1).

## Discussion

The possibility of implementing post-mortem diagnostics of mechanical asphyxia through increasingly early molecular markers of the hypoxic insult response is bound to a clear definition of the spectrum of the biological effects of miRNAs 26-27. The execution of the so-called molecular autopsy can, in cases where the diagnosis is difficult through the use of classical methods, represent an indisputable added value as it can demonstrate the vital reaction in its earliest stages according to high levels of evidence. Highlighting the cellular response to low oxygen concentrations undoubtedly represents a significant implementation of the diagnostics allowing to corroborate the evidence deriving from the study of the vitality of the lesions.

The evaluation of the upregulation and downregulation profiles of the selected miRNAs could provide useful interpretative elements for understanding the mechanisms involved in the acute response to hypoxia in asphyxia deaths. Nonetheless, the comparison between the expression profiles of hypoxamiRs and immunohistochemical markers of hypoxic stress on lung tissue could contribute to increasing the sensitivity and specificity in diagnosing the vitality of exposure to hypoxic insult 28-30.

Further, the stability of miRNAs offers significant research prospects in forensic pathology. In fact, the possibility of evaluating the molecular expression on formalin-fixed and paraffin-embedded (FFPE) samples allows the study of wide retrospective cases 31-33.

Given the defined diagnostic implications, it is essential to carry out further studies aimed at improving the knowledge in the field of miRNA biology and of the modalities of expression regulation in response to hypoxia 34,35. In particular, it is necessary to focus attention on the role of reactive oxygen species and self-regulation systems 36-37. Obviously, the diagnostic and forensic usefulness of molecular biology is linked to the overcoming of the limits related to the incomplete knowledge on the impact of the modulation of miRNA expression in response to low oxygen concentrations, but above all on the regulation of cell death programmed in response to hypoxia. In light of the evidence that emerged, it would be desirable to launch broad scientific research aimed at overcoming the knowledge gap in the field of hypoxamiRs regulation.

## Figures and Tables

**Table 1:** HypoxamiRs with a possible role in diagnostics of asphyxia deaths.

HypoxemiR	Expression	Regulatory effect
miR-20b 13	Downregulated	Derepression of HIF-1α
miR-26a 14	Upregulated	Pro‐apoptotic
miR-130 15	Upregulated	Enhancement of HIF-1α translation
miR-146b 16	Downregulated	Proinflammatory
miR-199a 17	Downregulated	Derepression of HIF-1α
miR-200b 18	Downregulated	Derepression of HIF-1α Induction of angiogenic response
miR-210 19-23	Upregulated	Mitochondrial functional damage Inhibition of DNA repair Cell cycle break, HIF-1α stabilization
miR-373 24	Upregulated	Inhibition of DNA repair
miR-424 25	Upregulated	HIF-1α stabilization
